# Imaging Amyloplasts in the Developing Endosperm of Barley and Rice

**DOI:** 10.1038/s41598-019-40424-w

**Published:** 2019-03-06

**Authors:** Ryo Matsushima, Hiroshi Hisano

**Affiliations:** Institute of Plant Science and Resources, Okayama University, Kurashiki, 710-0046 Japan

## Abstract

Amyloplasts are plant-specific organelles responsible for starch biosynthesis and storage. Inside amyloplasts, starch forms insoluble particles, referred to as starch grains (SGs). SG morphology differs between species and SG morphology is particularly diverse in the endosperm of Poaceae plants, such as rice (*Oryza sativa*) and barley (*Hordeum vulgare*), which form compound SGs and simple SGs, respectively. SG morphology has been extensively imaged, but the comparative imaging of amyloplast morphology has been limited. In this study, SG-containing amyloplasts in the developing endosperm were visualized using stable transgenic barley and rice lines expressing amyloplast stroma-targeted green fluorescent protein fused to the transit peptide (TP) of granule-bound starch synthase I (*TP-GFP*). The *TP-GFP* barley and rice plants had elongated amyloplasts containing multiple SGs, with constrictions between the SGs. In barley, some amyloplasts were connected by narrow protrusions extending from their surfaces. Transgenic rice lines producing amyloplast membrane-localized SUBSTANDARD STARCH GRAIN6 (SSG6)-GFP were used to demonstrate that the developing amyloplasts contained multiple compound SGs. *TP-GFP* barley can be used to visualize the chloroplasts in leaves and other plastids in pollen and root in addition to the endosperm, therefore it provides as a useful tool to observe diverse plastids.

## Introduction

Amyloplasts are a type of plastid surrounded by a double lipid bilayer of inner and outer envelope membranes^1^. Plants develop amyloplasts in storage organs such as the endosperm and tubers to biosynthesize and store glucose as starch. Starch is produced in the matrix space (stroma) of amyloplasts and forms particles referred to as starch grains (SGs), which exhibit different morphologies depending on the plant species. The morphological diversity is particularly marked in the endosperm of the Poaceae^2–8^. SG morphologies are classified as either compound or simple^9^. Compound SGs are formed by the assembly of small starch granules. In rice (*Oryza sativa*) endosperm, compound SGs normally develop to 10–20 μm in diameter, and are composed of individual sharp-edged polyhedral granules with a typical diameter of 3–8 μm; thus, the cross-sections of these compound SGs look like turtle shells^10^. In contrast, simple SGs are composed of single starch granules, and are found in barley (*Hordeum vulgare*), wheat (*Triticum aestivum*), and maize (*Zea mays*)^2,4^. Simple SGs are further divided into bimodal and uniform subtypes; the bimodal subtype comprises smaller and larger simple SGs, known as B-type and A-type SGs, respectively, which coexist in the same cells. The uniform subtype of simple SGs consists of similar-sized granules, which may be hexagonal, pentagonal, or round. Barley and wheat form bimodal SGs and maize produces uniform SGs. Phylogenetic studies have revealed that the compound type is ancestral and found in the majority of the Poaceae family, whereas the bimodal type is specific to a restricted cluster of genera within the Poaceae^2,3^. The mechanisms controlling the formation of compound and simple SGs have not yet been elucidated.

SGs can be stained violet by iodine to easily visualize their morphologies for standard light microscopy; however, iodine cannot be used to visualize the amyloplast itself, and therefore, amyloplast morphology must be investigated using other methods. For example, GFP-based imaging of amyloplasts has been used for both rice and wheat^11,12^. In both cases, the transit peptide (TP) of the granule-bound starch synthase I (GBSSI) was fused to the N-terminal end of GFP (TP-GFP). The TP causes the fused GFP to be transported to the surface of the amyloplasts and is cleaved off upon import of the fused GFP across the inner envelope membrane. The GFP can then be observed in the stroma of the amyloplasts and not inside of the starch granules, as it is unable to penetrate the tightly packed, semicrystalline glucan structure within the granules^11,12^. In wheat, confocal laser-scanning microscopy was used after the transient expression of *TP-GFP*^12^. This revealed that B-type SGs were located in the protrusions emanating from amyloplasts containing A-type SGs (A-type amyloplasts), and that these protrusions facilitated the interconnection of A-type amyloplasts. The similarity between the protrusions of the amyloplasts and the stromules of chloroplasts is noteworthy^13^. In the case of rice, some work has been done with stably transgenic plants expressing *TP-GFP*^11,14–16^. The typical dividing amyloplasts of rice are elongated, with multiple constrictions between SGs resulting in a beads-on-a-string structure^14^. It was suggested that amyloplast division progresses simultaneously at multiple sites^14^. As in the wheat amyloplasts, stromule-like protrusions were also observed in rice amyloplasts^14^.

Barley is another major crop in the Poaceae and is generally used for malting, food, and feed. Barley seeds accumulate starch mainly in the endosperm. Transgenic barley expressing *GFP* fused with full-length *GBSSI* cDNA (*GBSSI-GFP*) had been generated to observe SGs^17^. However, actual amyloplast had never observed in this transgenic barley, because GBSSI-GFP was incorporate into SGs. So far, no stable transgenic barley plants for visualizing amyloplasts had been previously reported. In this study, we generated stably transgenic barley and rice lines expressing amyloplast-targeted *GFP*, and used these to visualize the amyloplast morphologies of these two important crop species. We found that barley and rice amyloplasts contained multiple SGs during seed development, and we obtained clear images that are consistent with previous observations^12,14^. The transgenic plants generated in this study could be useful for comparing the amyloplast morphology in barley and rice.

## Results

### Morphology of SGs in barley endosperm

**Figure 1:**
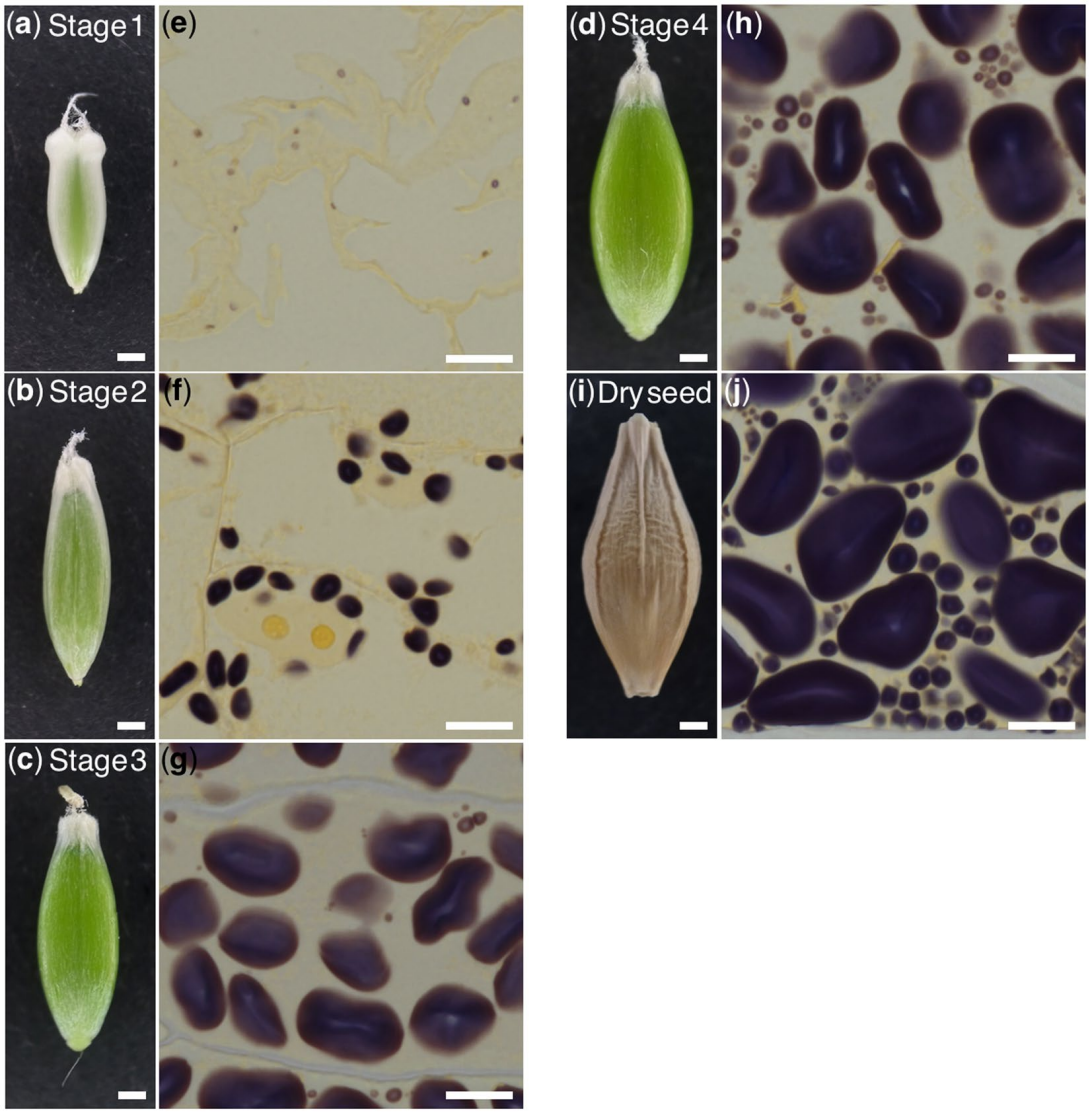
Development of starch grains in the endosperm of wild-type barley cv. Golden Promise. (**a**) Stage 1 seed. (**b**) Stage 2 seed. (**c**) Stage 3 seed. (**d**) Stage 4 seed. (**e**) Iodine-stained thin section of the seed in (**a**). (**f**) Iodine-stained thin section of the seed in (**b**). (**g**) Iodine-stained thin section of the seed in (**c**). (**h**) Iodine-stained thin section of the seed in (**d**). (**i**) Dry, mature barley seed. (**j**) Iodine-stained thin section of the seed in (**i**). Scale bars, 1 mm in (**a**–**d**,**i**); 10 μm in (**e**–**h**,**j**).

We observed barley amyloplast morphologies at different developmental stages. First, we tried to collect seeds at consecutive developmental stages based on the number of days after flowering (DAF). We used the barley cultivar Golden Promise, which is the most reliable haplotype for transformation^18^; however, under the growth conditions we used, Golden Promise often pollinates without obvious flower opening or heading. It was therefore difficult to determine the timing of pollination, and we could not collect the developing seeds based on DAF. Instead, we captured whole images of the developing seeds, classifying them into four categories (Stages 1 to 4) based on their lengths and widths (Supplementary Fig. 1). The seed length of Stage 2 and 3 were very similar, but seed width was different between these stages (Supplementary Fig. 1). In this study, we called Stage 1 and Stage 2 as early developing stages, and called Stage 3 and Stage 4 as later developing stages (Fig. 1a–d). We then embedded the seeds in Technovit resin for thin sectioning. Iodine staining of the sections allowed us to clearly visualize the developing SGs (Fig. 1e–h). At Stage 1, the SGs could be faintly detected as violet signals (Fig. 1e). At Stage 2, simple SGs appeared (Fig. 1f); most were similarly sized, and no bimodal size distribution was observed. The simple SGs became larger from Stage 2 to Stage 3 (Fig. 1g). Between Stage 3 and Stage 4, small and simple SGs appeared, resulting in a bimodal distribution of SG sizes (Fig. 1g,h). In older, dry seeds, the proportion of small and simple SGs further increased in comparison with Stage 4 (Fig. 1i,j).

### Visualization of barley amyloplast morphology in early developing endosperm

To visualize barley amyloplasts, we generated a stable transgenic barley plants expressing *GFP* fused with the TP of rice GBSSI (Supplementary Fig. 2a). These transgenic plants, referred to as *TP-GFP*, express the chimeric gene *TP-GFP* under the control of the maize *Ubiquitin 1* promoter.

**Figure 2:**
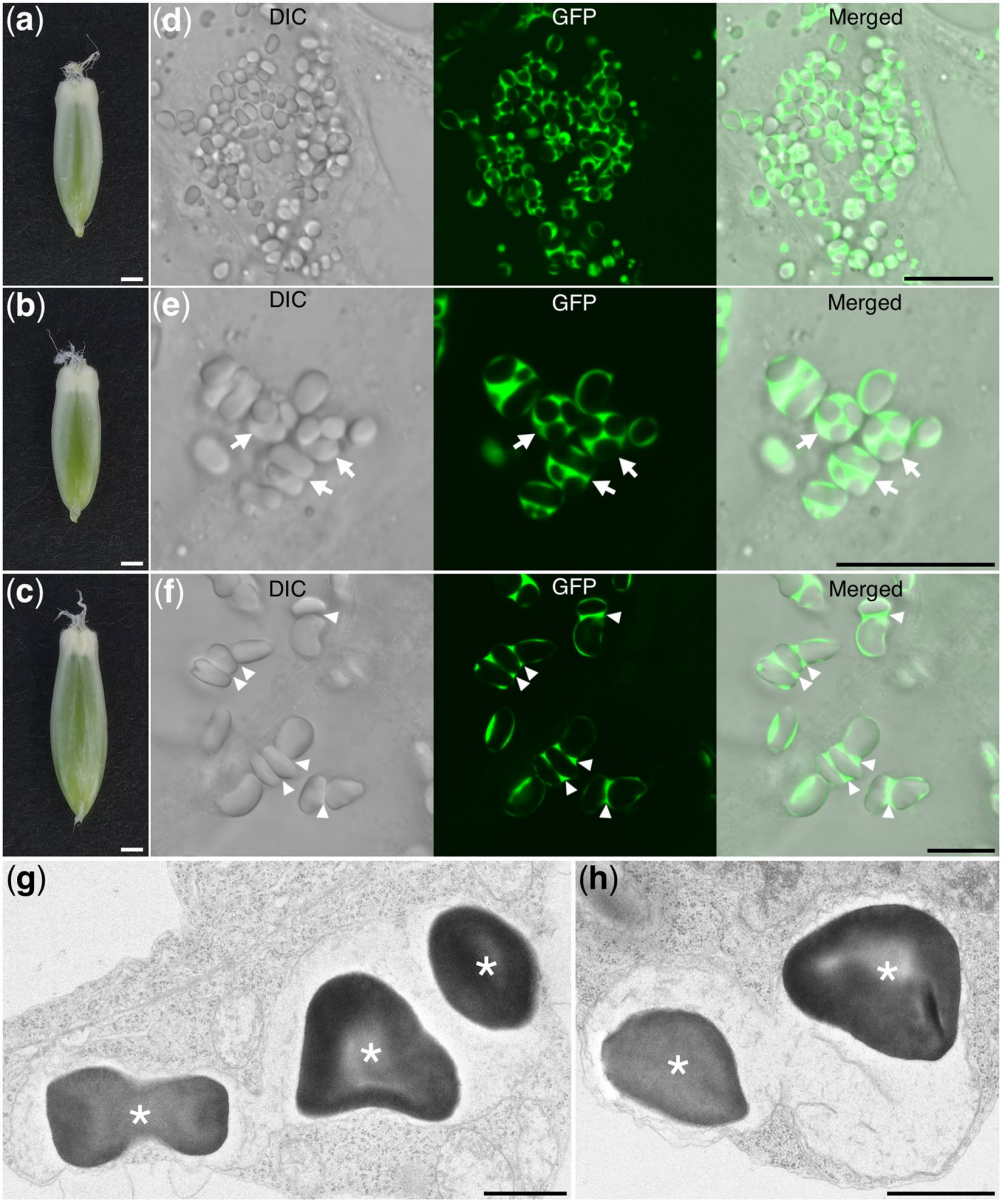
Fluorescence images of the endosperm at Stages 1 and 2 in transgenic *TP-GFP* barley. (**a**,**b**) Stage 1 seeds. (**c**) Stage 2 seed. (**d**) Differential interference contrast (DIC), GFP, and merged images of the section prepared from (**a**). (**e**) DIC, GFP, and merged images of the section prepared from (**b**). Arrows indicate amyloplasts containing multiple starch grains (SGs). (**f**) DIC, GFP, and merged images of the section prepared from (**c**). Arrowheads indicate GFP-enriched regions inside amyloplasts. (**g**,**h**) Transmission electron micrographs of amyloplasts in non-transgenic barley endosperm. Asterisks indicate SGs. Scale bars, 1 mm in (**a**–**c**); 10 μm in (**d**–**f**); 1 μm in (**g**,**h**).

To examine the amyloplasts, we first prepared slices from the early developing seeds of *TP-GFP* barley (Fig. 2a–c). A plot of the sizes of each developing seed (Supplementary Fig. 1) showed that these were similar in size to the wild-type Stage 1 and Stage 2 seeds shown in Fig. 1. Next, we examined the central parts of the seed endosperm using confocal laser-scanning microscopy and obtained differential interference contrast (DIC) and GFP images. In the DIC images, the simple SGs were visible as particles (Fig. 2d–f, left panels). GFP was transported into the stroma of the amyloplasts and clearly revealed the amyloplast morphology (Fig. 2d–f, middle panels). In Stage 1 endosperm, the merged DIC and GFP images showed that the GFP signals surrounded the simple SGs in the amyloplasts (Fig. 2d,e; right panels), and the SGs were observed as black spaces in the amyloplasts in the GFP images (Fig. 2e; middle panel). In most cases, an amyloplast contained multiple simple SGs. In Stage 2, the SGs became larger and the amyloplasts were more elongated than those in Stage 1 (Fig. 2f). Following the growth of SGs, the GFP signals surrounded the SGs became thinner (Fig. 2f, middle panel), and GFP between the SGs within the amyloplasts seemed like concentrated (Fig. 2f, arrowheads). Similar images were obtained in the Stage 2 samples (n = 3 biological replicates, Supplementary Fig. 3). This result suggested that each individual amyloplast contained multiple simple SGs in Stage 1 and Stage 2 seeds. Observations using transmission electron microscopy (TEM) also showed that wild-type barley developed amyloplasts containing multiple simple SGs (Fig. 2g,h). This result excluded the possibility that the expression of *GFP* caused the amyloplasts to contain multiple SGs. Occasionally, the cylindrical GFP signals that interconnect SGs were also observed at Stage 1 (Supplementary Fig. 4).

### Visualization of barley amyloplast morphology in later developing endosperm

**Figure 3:**
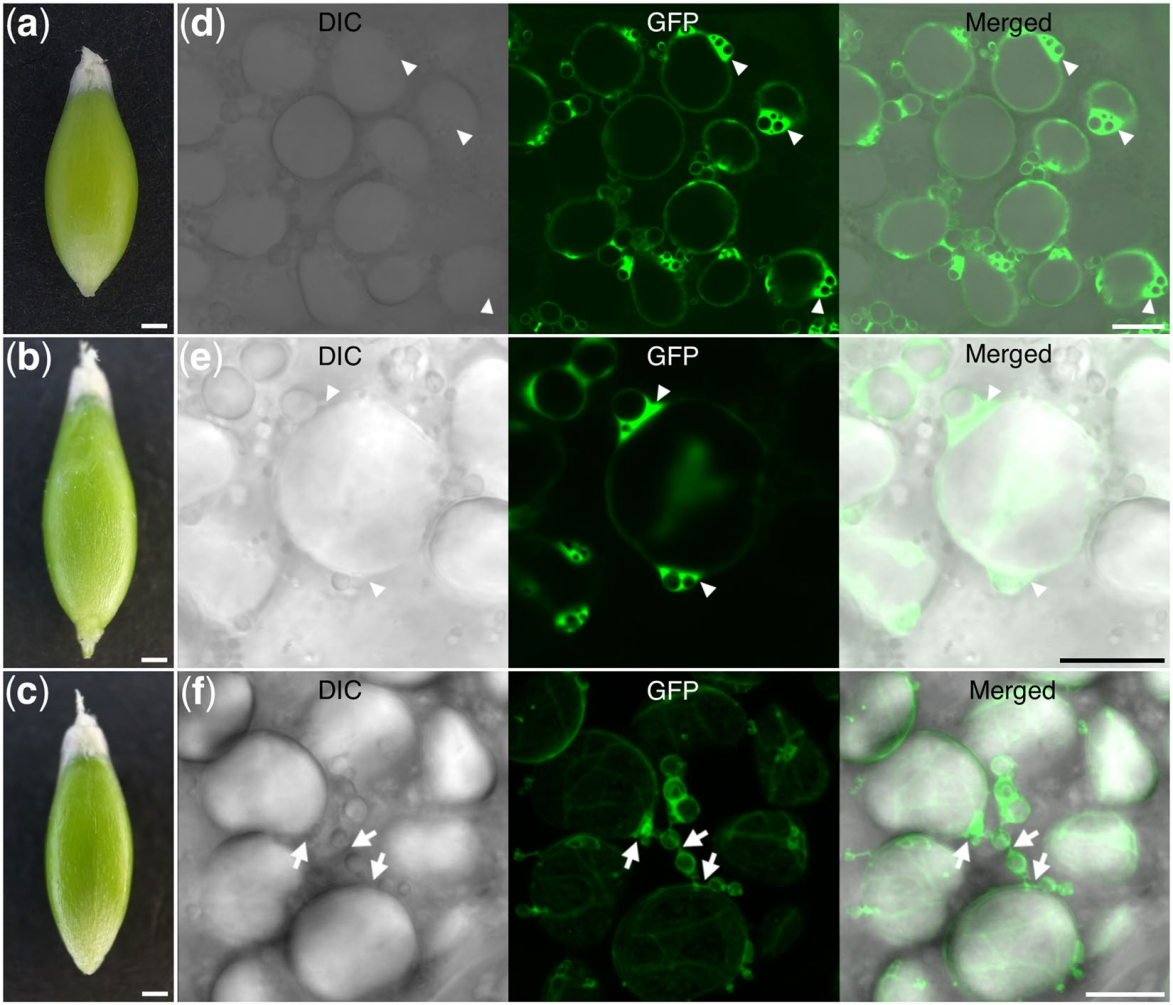
Fluorescence images of endosperm between Stage 3 and Stage 4 in transgenic *TP-GFP* barley. (**a**–**c**) The seeds between Stage 3 and Stage 4. (**d**,**e**) Differential interference contrast (DIC), GFP, and merged images of the sections prepared from (**a**,**b**), respectively. Arrowheads indicate GFP-enriched regions. (**f**) Projections of seven confocal optical sections taken at 0.42-μm intervals along the *z* axis. The sections were prepared from the seed in (**c**). Amyloplasts containing B-type SGs are connected by cylindrical GFP-localized regions. Cylindrical GFP regions also connect B-type to A-type amyloplasts. Arrows indicate cylindrical GFP-labeled regions. Scale bars, 1 mm in (**a**–**c**); 10 μm in (**d**–**f**).

We next observed amyloplasts in later stages of endosperm development. Because the small (B-type) SGs appeared between Stage 3 and Stage 4 (Fig. 1g,h), observation between these stages would be suitable to observe the dynamic changes of SG morphologies. We prepared slices from seeds between Stage 3 and Stage 4 (Fig. 3a–c, Supplementary Fig. 1). Using DIC, we observed both of A- and B-types of SGs (Fig. 3d,e, left panels). GFP signals surrounded the SGs (Fig. 3d,e, middle panels), and the GFP-labeled stroma was present in the region between the A-type and B-type SGs (Fig. 3d,e, arrowheads). A *z*-projection of confocal slices showed that amyloplasts containing B-type SGs were connected to each other by cylindrical GFP signals (Fig. 3f, arrows; Supplementary Movie 1). In Supplementary Movie 1, 3D image obtained from the stack of the seven confocal images used in Fig. 3f. Tilting of the 3D image was shown. B-type SGs were also connected to the amyloplasts containing A-type SGs. These observations indicate that A-type and B-type simple SGs were present in the same amyloplasts between Stage 3 and Stage 4.

### Morphology of SGs in rice endosperm

**Figure 4:**
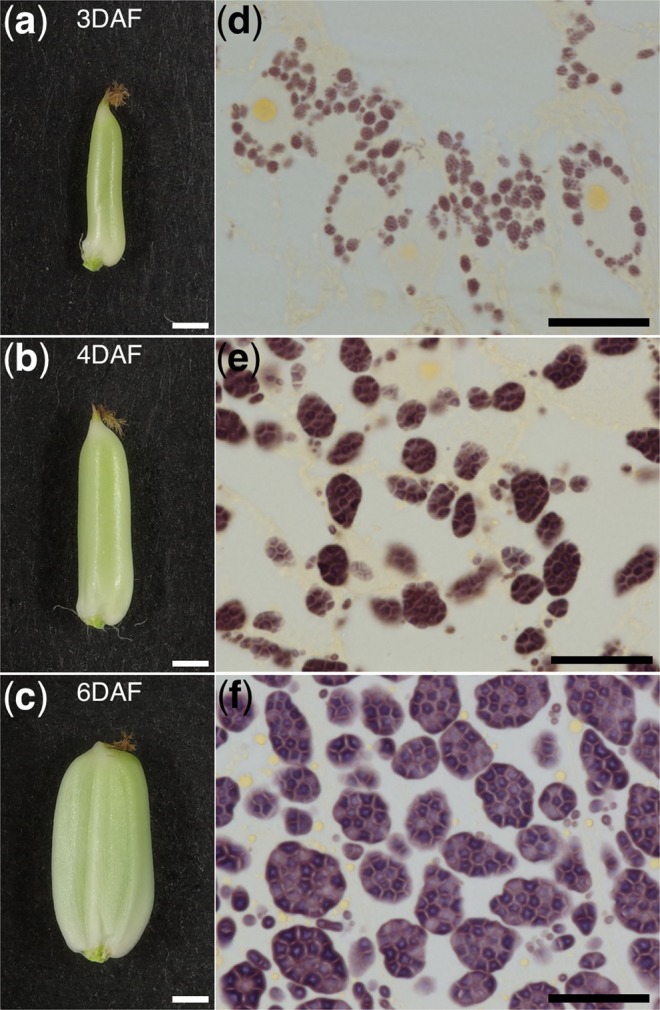
Starch grains in developing endosperm of wild-type rice cv. Nipponbare. (**a**) Rice seed at 3 DAF. (**b**) Rice seed at 4 DAF. (**c**) Rice seed at 6 DAF. (**d**) Iodine-stained thin section of endosperm at 3 DAF. (**e**) Iodine-stained thin section of endosperm at 4 DAF. (**f**) Iodine-stained thin section of endosperm at 6 DAF. Scale bars, 1 mm in (**a**–**c**); 20 μm in (**d**–**f**).

We also examined SGs and amyloplasts from rice using the cultivar Nipponbare. In contrast to barley, flower opening in rice is clearly evident, so the DAF times were easy to determine. We collected developing seeds at 3, 4, and 6 DAF and used them to prepare Technovit thin sections (Fig. 4a–c). We also observed endosperm at 2 DAF (data not shown), but found that endosperm development at this point was insufficient for the observation of SGs using iodine staining. At 3 DAF, small compound SGs were clearly observable in the central part of the endosperm (Fig. 4d). From 3 to 6 DAF, the compound SGs enlarged dramatically (Fig. 4d–f).

### Visualization of amyloplast morphology in developing rice endosperm

**Figure 5:**
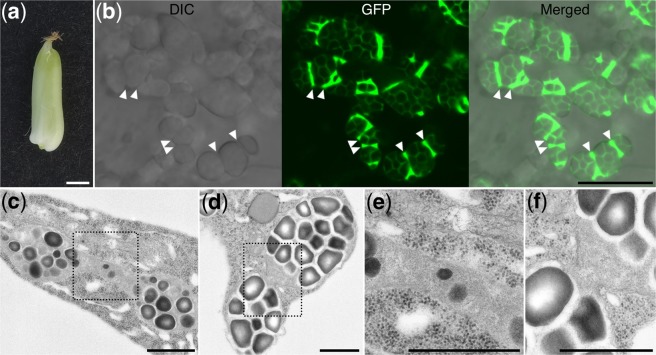
Fluorescence images of the endosperm in transgenic *TP-GFP* rice. (**a**) Rice seeds at 4 DAF. (**b**) Differential interference contrast (DIC), GFP, and merged images of the sections prepared from (**a**). Arrowheads indicate GFP-enriched regions inside amyloplasts. (**c**,**d**) Transmission electron micrographs of amyloplasts in the non-transgenic rice endosperm. (**e**,**f**) Magnified images of areas indicated by dotted lines in (**c**,**d**), respectively. Constrictions of amyloplasts are visible. Scale bars, 1 mm in (**a**); 10 μm in (**b**); 1 μm in (**c**–**f**).

We generated transgenic *TP-GFP* rice using the same vector used to create the transgenic barley (Supplementary Fig. 2a). We prepared slices from 4-DAF seeds (Fig. 5a), as the seeds at 3 DAF were too small to prepare slices by hand and the seeds at 6 DAF were not suitable for preservation of the integrity of the amyloplast membrane. DIC images of the central part of the endosperm revealed the presence of compound SGs (Fig. 5b, left panel). The GFP localized between the starch granules in the compound SGs in a net-like structure (Fig. 5b, middle panel), and the GFP-labeled stroma was enriched in the regions between the compound SGs (Fig. 5b, arrowheads). This indicates that multiple compound SGs were present in a single amyloplast. Amyloplasts containing two compound SGs were confirmed using TEM observations in non-transgenic rice endosperm (Fig. 5c,d). Magnified TEM images showed the constriction of the amyloplasts (Fig. 5e,f).

**Figure 6:**
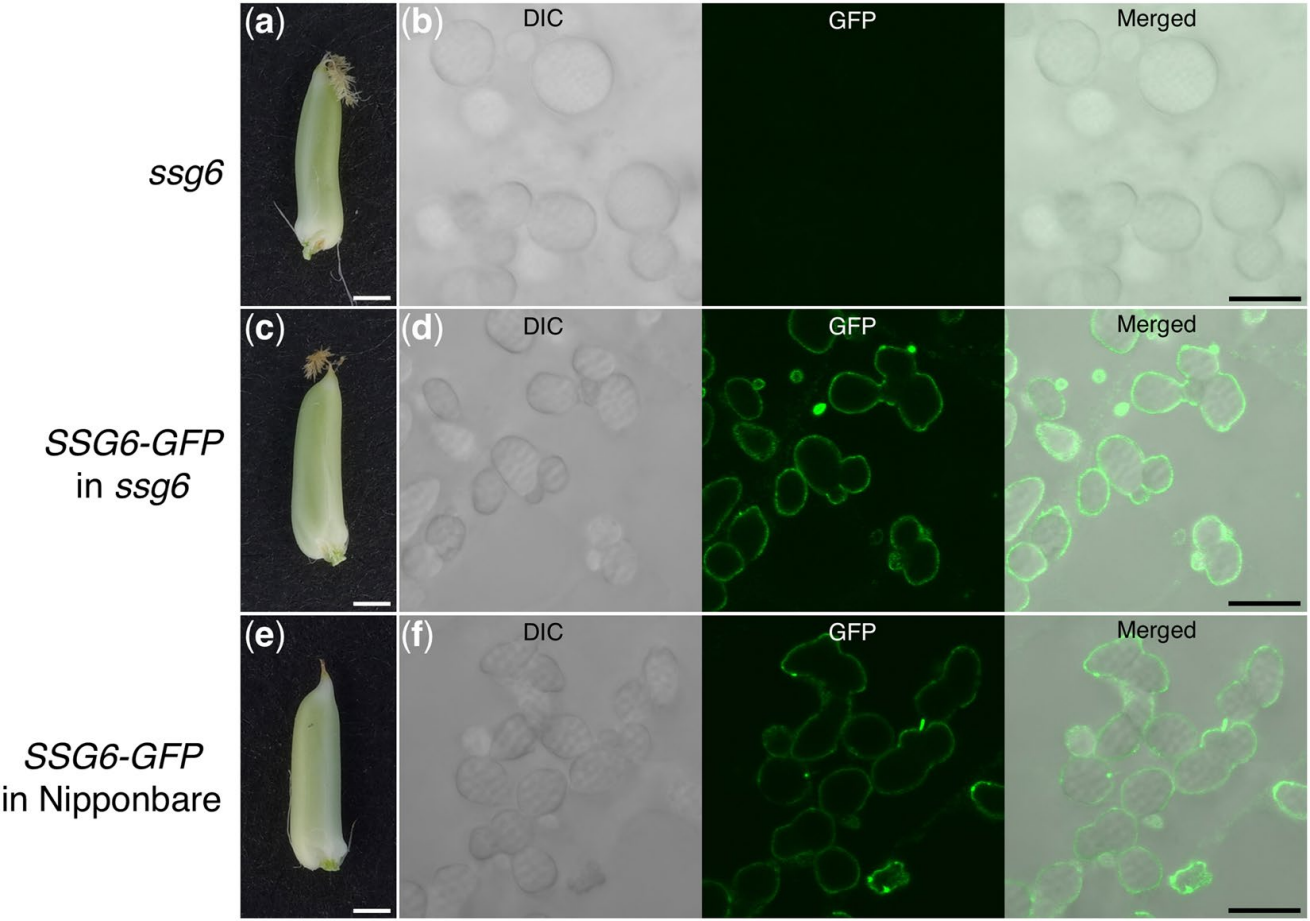
Fluorescence images of the endosperm in transgenic rice expressing *SSG6-GFP*. (**a**) Seed of the non-transgenic *ssg6* mutant at 4 DAF. (**b**) Differential interference contrast (DIC), GFP, and merged images of the section prepared from the seed in (**a**). (**c**) Seed of *ssg6* plant expressing *SSG6-GFP* at 4 DAF. (**d**) DIC, GFP, and merged images of the section prepared from seed in (**c**). (**e**) Seed of wild-type plant expressing *SSG6-GFP* seeds at 4 DAF. (**f**) DIC, GFP, and merged images of the section prepared from seed in (**e**). Scale bars, 1 mm in (**a**,**c**,**e**); 10 μm in (**b**,**d**,**f**).

SUBSTANDARD STARCH GRAIN6 (SSG6) is an amyloplast-membrane-localized protein in rice^19^. SSG6 is thought to control the size of compound SGs, as the *ssg6* mutant developed enlarged, spherical compound SGs (Fig. 6a,b)^19^. We previously constructed a plasmid expressing *SSG6* fused with *GFP*^19^ (Supplementary Fig. 2b). When we introduced the *SSG6-GFP* fusion into the *ssg6* mutant (*SSG6-GFP* in *ssg6*), the SGs were smaller than those of the parental *ssg6* line (Fig. 6c,d, left panels; Supplementary Fig. 5). In addition, the size and shape of SGs were similar between *SSG6-GFP* in *ssg6* and *SSG6-GFP* in Nipponbare rice (Fig. 6e,f, left panels; Supplementary Fig. 5), indicating that SSG6-GFP was functional. GFP imaging indicated that SSG6-GFP localized to the outer limit of the amyloplast membrane (Fig. 6d,f, middle panels). The GFP signal surrounded the multiple compound SGs, indicating that amyloplasts contain more than one compound SG during seed development.

### Visualization of plastids in other organs in barley and rice

**Figure 7:**
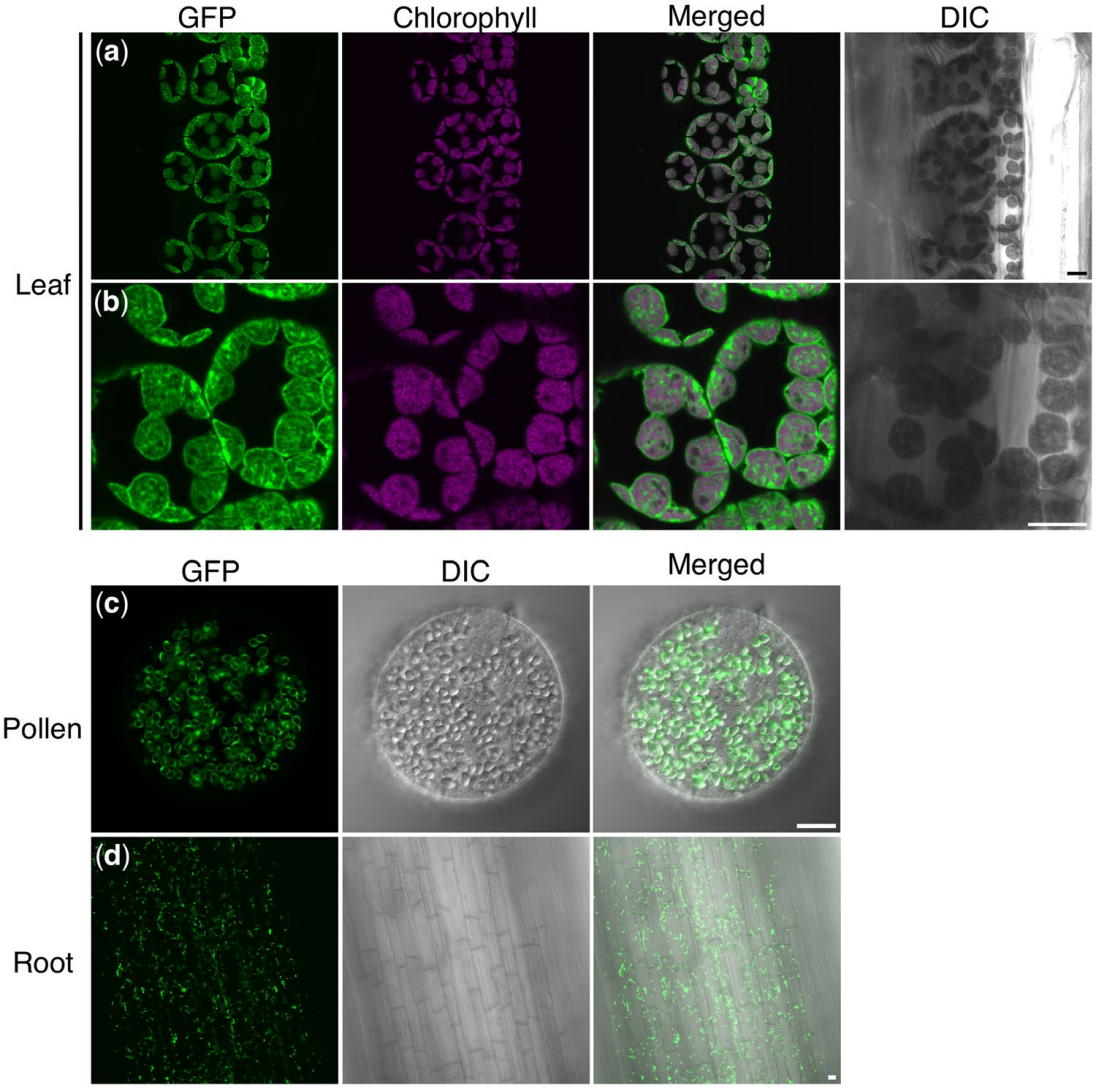
Fluorescence images of chloroplasts in leaves, plastids in pollen and root of *TP-GFP* barley. (**a**,**b**) GFP and chlorophyll autofluorescence and merged images of *TP-GFP* barley leaves. Differential interference contrast (DIC) image was also shown. (**c**) GFP, DIC and merged images of *TP-GFP* barley pollen. (**d**) GFP, DIC and merged images of *TP-GFP* barley root. Scale bars, 10 μm.

We were able to clearly visualize chloroplasts in the leaves, amyloplasts in the pollen grains, and plastids in the roots of *TP*-*GFP* barley (Fig. 7a–d). In non-transgenic barley plants, no GFP signal was detected under the same detection condition and similar chlorophyll images in leaf and rod-like SGs in pollen grains were observed (Supplementary Fig. 6). In the case of the *TP-GFP* rice, GFP-labeled amyloplasts in pollen grains were also clearly visualized like endosperms (Supplementary Fig. 7), however we could not obtain clear GFP-images in leaves and roots (data not shown).

## Discussion

In this study, we visualized amyloplasts containing SGs using stroma-localized GFP in transgenic barley and rice. We showed that GFP fused with transit peptide of rice GBSSI was functional in barley. This is the first example to develop the stable transgenic plants visualizing amyloplasts in simple-SG-developing plants.

In the developing endosperms of barley and rice, we observed multiple SGs inside individual amyloplasts (Figs 2, 3, 5 and 6). A previous study in transgenic rice had shown that amyloplast division progresses simultaneously at multiple sites and that elongated amyloplasts containing multiple compound SGs undergo several constrictions simultaneously^14^. Those results resemble the phenomena we observed in barley and rice, and we therefore concluded that the amyloplasts containing multiple SGs are amyloplasts in the process of division.

In barley, we observed B-type small SGs in the peripheral stroma and GFP-labeled protrusions of the A-type amyloplasts (Fig. 3d–f, middle panels). Similar images were obtained from wheat endosperm transiently expressing *TP-GFP*^12,20^. Considering these similarities, it seems likely that the above characteristics are common features of amyloplasts in species biosynthesizing bimodal SGs, such as barley and wheat. In rice, the budding-type amyloplast division in which large amyloplasts divide by protrusion of small amyloplasts from the surface were also observed^14^. Our observation of B-type small SGs in the peripheral stroma and the protrusions from the A-type amyloplasts might be comparable to the rice budding-type amyloplast division. The B-type amyloplasts was budding from A-type amyloplasts and the cylindrical structure might be intermediate process of the budding process of B-type amyloplasts from the A-type. Even though SG morphologies were different between rice and barley, the mechanism of amyloplast division process may be common.

Previous observation of the transgenic rice expressing *TP-GFP* focused on the lateral side of the endosperm (i.e., the subaleurone cells)^11,14^. The amount of the SGs was less in the lateral part compared to the central part of the endosperm in rice seeds^10^. In the endosperm of 4-DAF seeds, amyloplasts in the lateral side of the endosperm was small and sparse (Supplementary Fig. 8). Therefore, we observed SG at central part in which SGs could be much more developed. Consistent with previous observation^11,14^, amyloplast stroma-targeted GFP does not penetrate into the starch granules and therefore localizes around them, where it is observable as a net-like structure in the compound SGs (Fig. 5b). The degree of constriction of amyloplasts in this study was minimal compared to the previous observation of rice amyloplasts in which the constriction was remarkable. This may be because the endosperm was observed at an earlier point in the division process in this study, compared to the previous one^16^.

To visualize the amyloplast membrane, we introduced the *SSG6-GFP* gene into wild-type and *ssg6* mutant rice (Fig. 6). SSG6 has a putative transmembrane domain and homology to aminotransferases^19^. The *ssg6* mutant develops enlarged, spherical compound SGs in the endosperm (Fig. 6b, left panel). In this study, SSG6-GFP localized at the amyloplast membrane in the *ssg6* mutant background (Fig. 6d, middle panel). In a previous study, the fusion protein of an ADP-glucose transporter (Brittle1, BT1) and GFP (BT1-GFP) localized to the outer edge of the amyloplasts, as well as between the granules of the compound SGs^16^. BT1 is an inner envelope membrane protein^21^ indicating the presence of an inner envelope-containing structure between the granules^16^. In contrast, SSG6-GFP was only detected at the outer edge of the amyloplasts (Fig. 6d,f, middle panels), and may therefore be localized at the outer rather than the inner envelope membrane.

We previously developed a simple method for observing SGs in rice^10^. Using this method, we isolated rice mutants (*ssg*) with defective SG morphologies. The *ssg1*, *ssg2*, and *ssg3* mutants had higher numbers of smaller SGs (<10 μm in diameter) in addition to the normal-sized SGs (10–20 μm in diameter). Also, *ssg4* and *ssg6* developed enlarged compound SGs (>30 μm in diameter), while the SGs of *ssg5* lacked the characteristic compound SG structure^10,19,22^. We are now in the process of isolating barley mutants with defective SG morphologies using the same methods as were described for rice. Crossing these barley mutants with the transgenic *TP-GFP* barley line generated in this study will allow us to characterize the structure of the amyloplasts in the mutants, which may help elucidate the way in which amyloplast morphology is related to the SG defects in the mutants.

Chloroplasts in leaves and plastids in roots had been clearly visualized in rice by the expression of *GFP* fused with transit peptide of Rubisco Small Subunit2 under the control of CaMV 35S promoter previously^23^. In this paper, other organs except for leaves and roots were observed. In the transgenic *TP*-*GFP* plants in this study, *TP*-*GFP* was under the regulation of the maize *Ubiquitin 1* promoter, therefore, we expected the GFP could be visualized throughout the plants. However, we could not obtain GFP signals in leaves and roots in the T1 transgenic rice. This might be because gene silencing of *TP-GFP* was occurred specifically at leaves and roots. On the other hand, the transgenic barley plants developed in this study showed GFP-labeled chloroplast and other plastids in leaves, pollens, and roots as well as endosperm (Fig. 7) even after T2 generation. Therefore, this transgenic barley could be used for the further analysis of amyloplasts in endosperm, but also for the study of other types of plastids in whole organs.

## Methods

### Plant material and growth conditions

Rice (*Oryza sativa* cv. Nipponbare) was grown at 28 °C (13-h day/11-h night for the first month then 11-h day/13-h night) in a growth cabinet (#LPH-411S; NK Systems, Japan). Barley (*Hordeum vulgare* cv. Golden Promise) was grown at 15 °C/13 °C (12-h day/12-h night for the first two months then 16-h day/8-h night) in a growth room. Rice *SSG6-GFP* plants were constructed as described previously^19^.

### Plasmid construction and transformation

To visualize amyloplasts, a vector was generated containing enhanced *GFP* (*EGFP*) attached to the sequence of a transit peptide targeted to the amyloplasts, under the regulation of the maize *Ubiquitin 1* promoter. The plasmid containing *EGFP* downstream of the maize *Ubiquitin 1* promoter, pBUH3-EGFP, was described in a previous study^24^. First, the nucleotide sequence encoding the transit peptide of *GRANULE-BOUND STARCH SYNTHASE I* (*Os06g0133000*) was amplified from the genome using the following primers: 5′-GTTACTTCTGCAGGGATGTCGGCTCTCACCACG-3′ and 5′-GCTCACCATGGTGGGGGTGGCGTACACGACGAC-3′ (the vector-derived sequence is underlined). The obtained *Os06g0133000* fragment encodes a portion of the protein from the first methionine to the 79th amino acid. The fragment was cloned into the *Sac*I sites of pBUH3-EGFP. The resulting plasmid vector was named pBUHWxTpGFP and used for *Agrobacterium tumefaciens*-mediated transformation in barley and rice, as described previously^25,26^.

### Preparation of Technovit sections from endosperm

To prepare thin sections of endosperm from dry seeds, approximately 1-mm^3^ blocks were dissected from the central region of the endosperm and fixed in FAA solution (5% [v/v] formalin, 5% [v/v] acetic acid, and 50% [v/v] ethanol) for at least 12 h at room temperature. To prepare thin sections of developing endosperm, approximately 1-mm^3^ blocks were dissected from the endosperm and fixed in 3% (v/v) glutaraldehyde in 20 mM cacodylate buffer (pH 7.4) for at least 24 h at 4 °C. The method of resin embedding using Technovit 7100 resin (Kulzer, Germany) was described previously^27^. Thin sections (1 μm) were prepared using an ultramicrotome (LEICA EM UC7; Leica Microsystems, Germany) and diamond blades. The sections were stained as described previously^27^.

### Transmission electron microscopy

Developing endosperm was fixed overnight in 4% (w/v) paraformaldehyde, 2% (v/v) glutaraldehyde in 50 mM cacodylate buffer (pH 7.4) at 4 °C, then post-fixed with 2% osmium tetroxide for 3 h at 4 °C. The methods used to prepare ultra-thin sections (80–90 nm) were described previously^22^. The samples were observed using a transmission electron microscope (JEM-1400Plus; JEOL, Japan) at an acceleration voltage of 80 kV. Digital images were captured using a CCD camera (EM-14830RUBY2; JEOL).

### Detection of GFP signals in endosperms of transgenic plants

Developing seeds without husks were set in elderberry pith (#1-150-0470; KENIS, Japan) and places in a hand microtome (#3-150-0461; KENIS). Slices were dissected from the fixed seeds using straight razors (#BTM-10H1; KAI Group, Japan). The obtained slices were placed on a glass slide, immersed in a drop of 550 mM sorbitol solution, and covered with a coverslip. GFP signals were detected using a laser-scanning confocal microscope (FV1000; Olympus Corporation, Japan).

### Quantification of images

The micrograph images were quantified using Fiji image-processing software^28^. DIC images were used for the quantification of the SG areas. To discard the SGs oriented in a non-sagittal plane, only the SGs with the two largest areas in each micrograph were used for the quantification.

### Observation of leaves, pollen grains and roots

To observe chloroplasts in leaves, approximately 1-mm strips were detached from the third leaves of barley seedling, placed on a glass slide, and immersed in a drop of water. After being covered with a coverslip, the leaves were examined with the laser-scanning confocal microscope. To observe amyloplasts in pollens, anthers just before anthesis were disrupted with forceps in water on a glass slide and released pollen grains were examined with the laser-scanning confocal microscope. Roots of elongation zone at 4 days after germination were examined like leaves.

## Supplementary information

Supplementary Figure 1-8

Supplementary Movie 1.
