# Supplementary material for: Imaging Amyloplasts in the Developing Endosperm of Barley and Rice
Source: Sci Rep. 2019 Mar 6;9:3745. doi: 10.1038/s41598-019-40424-w (PMC6403327; doi:10.1038/s41598-019-40424-w)
Supplement: Supplementary file 1 — Supplementary Figure 1-8 [file 41598_2019_40424_MOESM1_ESM.pdf]

Imaging Amyloplasts in the Developing Endosperm of Barley and Rice

Ryo Matsushima and Hiroshi Hisano

Institute of Plant Science and Resources,
Okayama University, Kurashiki, 710-0046, Japan

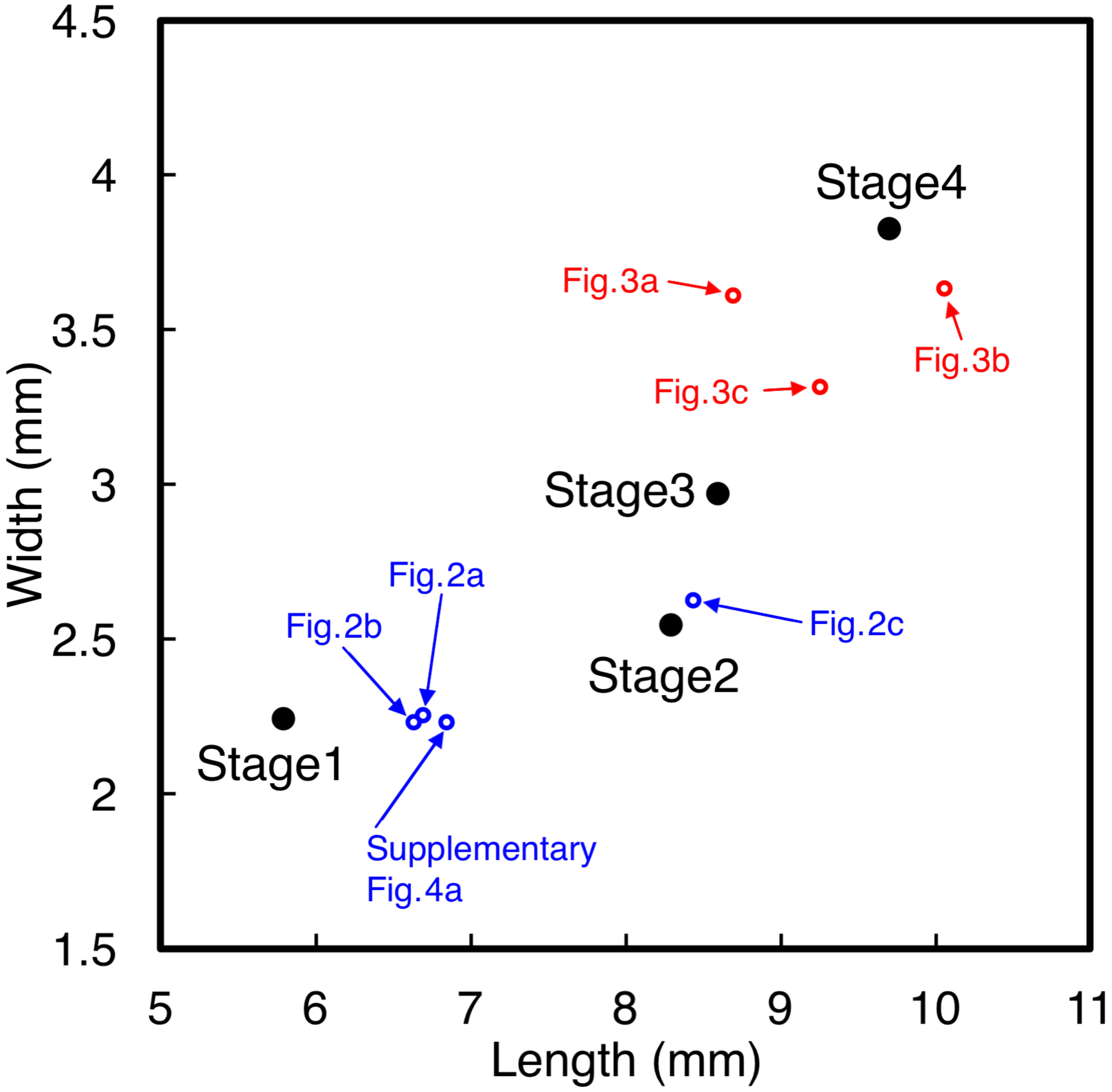

Supplementary Figure 1. Length and width of developing seeds at Stages 1-4. Black spots indicate the developing seeds of each stage in Figure 1, blue spots from Figure 2 and Supplementary Figure 4, and red spots from Figure 3.

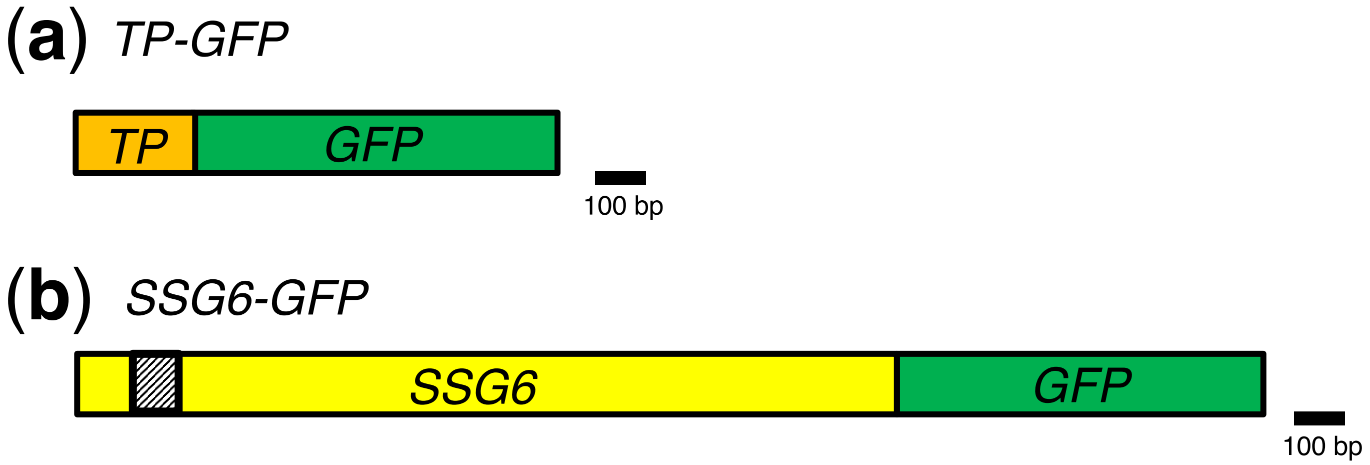

Supplementary Figure 2. Chimeric genes used for the construction of *TP-GFP* and *SSG6-GFP* plants. (**a**) Schematic view of the chimeric gene *TP-GFP*, encoding GFP fused to the transit peptide (TP) of the granule-bound starch synthase I. (**b**) Schematic view of the chimeric gene *SSG6-GFP*, encoding GFP fused to SSG6. The shaded box indicates the putative transmembrane domain. In the transgenic plants, *TP-GFP* and *SSG6-GFP* are expressed under the regulation of the maize *Ubiquitin 1* promoter.

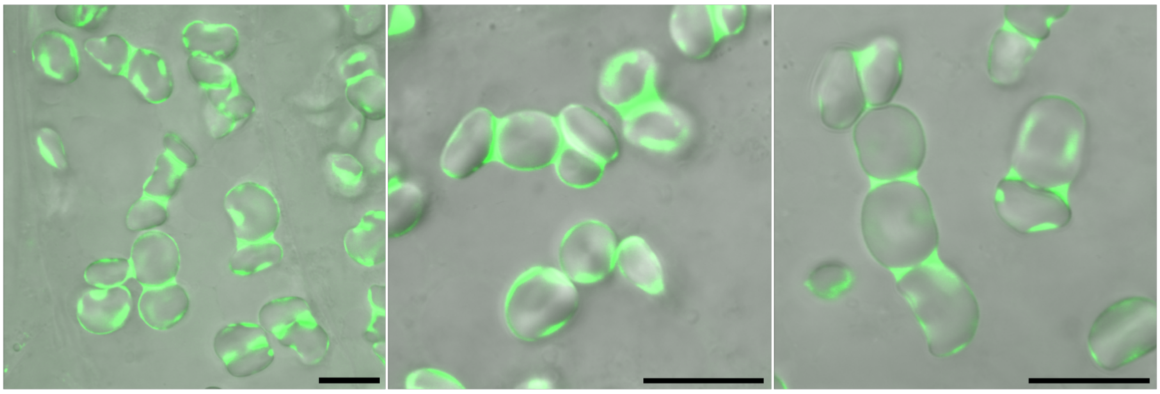

Supplementary Figure 3. Merged GFP and differential interference contrast images from slices prepared from early developing *TP-GFP* barley seeds. The images were obtained from three different seeds. The amyloplasts contain multiple starch grains. Scale bars, 10 µm.

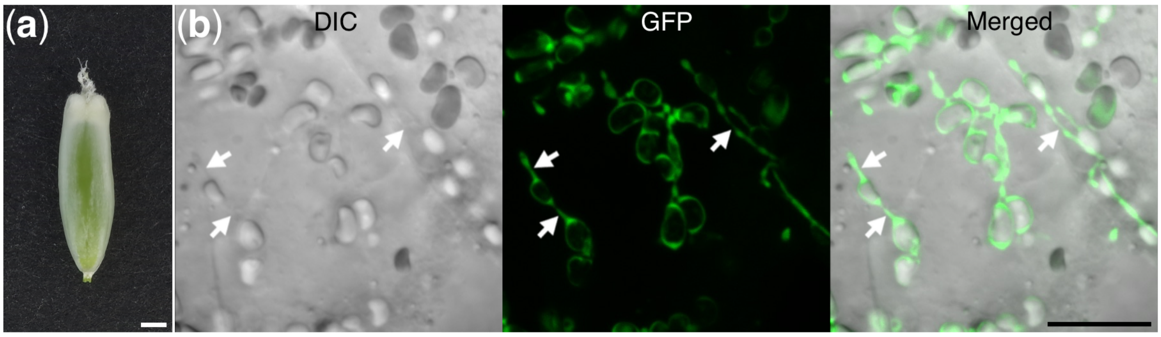

Supplementary Figure 4. Cylindrical GFP signals that interconnect SGs. (**a**) Stage 1 seed. (**b**) Differential interference contrast (DIC), GFP, and merged images of the sections prepared from (**a**). Six confocal optical sections were taken at 0.42-µm intervals along the *z* axis. Arrows indicate stromule-like structures of amyloplasts. Scale bars, 1 mm in a; 10 µm in b.

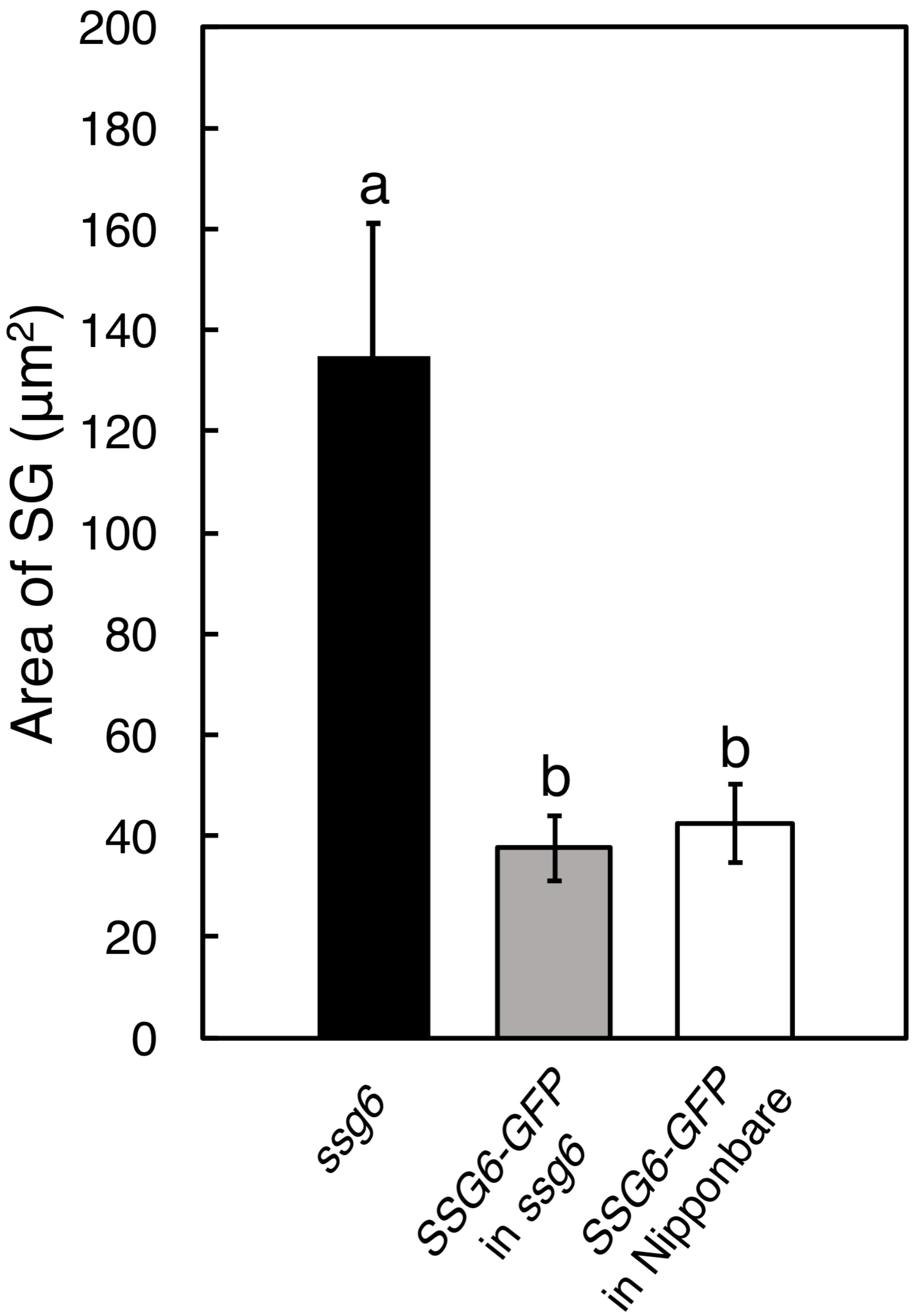

Supplementary Figure 5. Quantification of the SG areas in *ssg6*, *ssg6* plants expressing *SSG6-GFP*, and wild-type (Nipponbare) rice expressing *SSG6-GFP* (n = 8 each). Data are given as means ± SD. Statistical comparisons were performed using a Tukey test. Different letters represent statistically significant differences ($p < 0.01$).

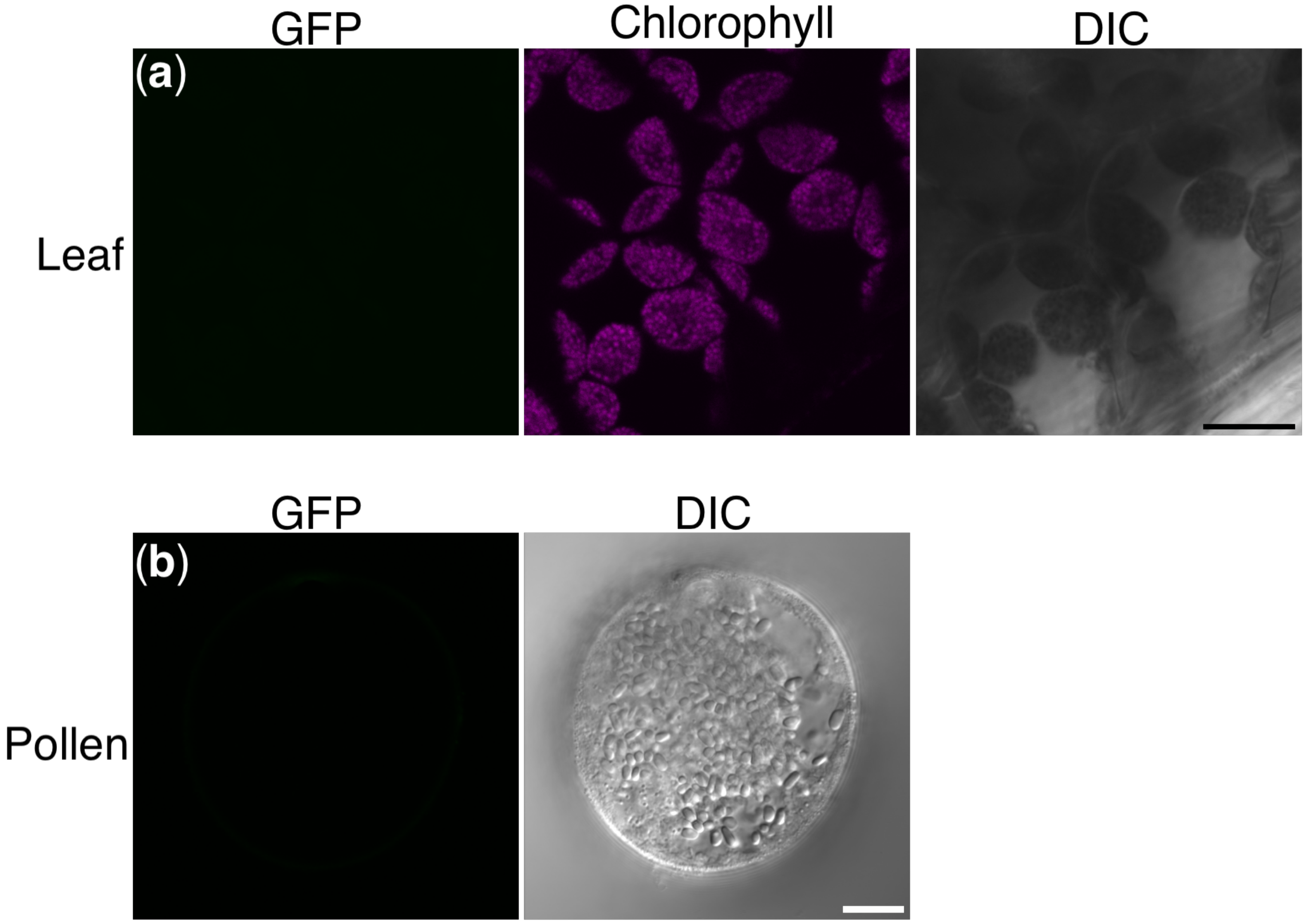

Supplementary Figure 6. Fluorescence images of leaf and pollen of non-transgenic barley. (**a**) GFP and chlorophyll autofluorescence and differential interference contrast (DIC) images of non-transgenic barley leaf. (**b**) GFP and DIC images of non-transgenic barley pollen. In both of leaf and pollen, no GFP signal was detected under the same detection condition as Figure 7. Scale bars, 10 µm.

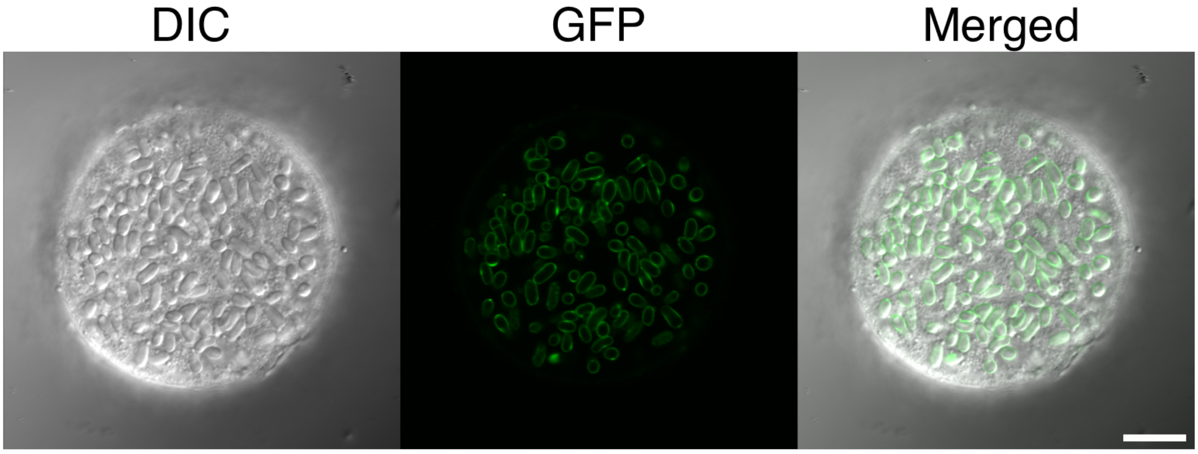

Supplementary Figure 7. Fluorescence images of pollen from *TP-GFP* rice. Differential interference contrast (DIC), GFP, and merged images of *TP-GFP* rice pollen. Scale bars, 10 µm.

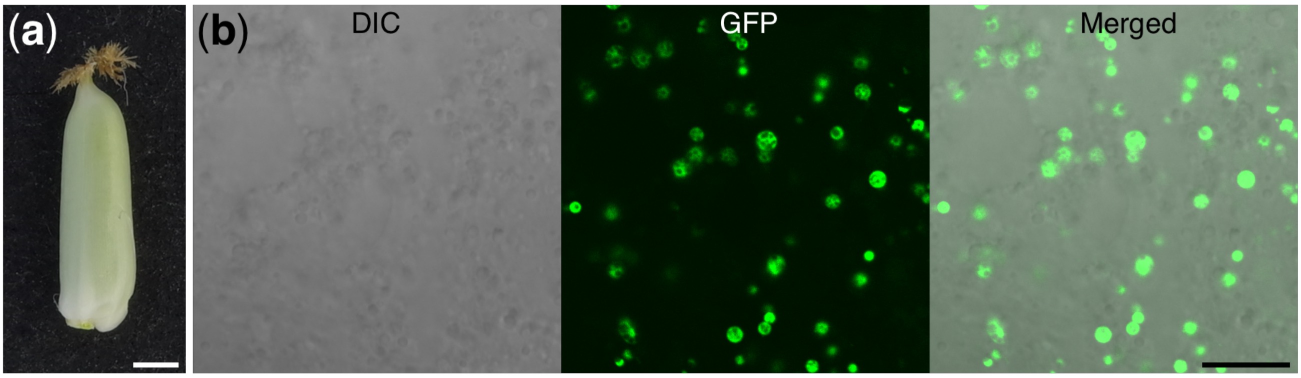

Supplementary Figure 8. Fluorescence image of the lateral side of the endosperm in transgenic *TP-GFP* rice. (**a**) Rice seed at 4 DAF. (**b**) Differential interference contrast (DIC), GFP, and merged images of the sections prepared from (**a**). Amyloplasts in the lateral side of the endosperm was small and sparse*.* Scale bars, 1 mm in a; 10 µm in b.
